# Risk Stratification Tools and Scoring Systems in Perforated Viscus: A Systematic Review

**DOI:** 10.7759/cureus.93101

**Published:** 2025-09-24

**Authors:** Murhaf Assaf, Shrey K Solanki, Ridham N Patel, Kinza Ali, Miqdad Qandeel, Nimrah Majeed, Shabeh E Roshan Ali, Mazin Osman, Kiranjot Kaur, Shahid Khan

**Affiliations:** 1 General Surgery, Princess of Wales Hospital, Cwm Taf Morgannwg University Health Board, Bridgend, GBR; 2 Emergency Medicine, Jivraj Mehta Hospital, Ahmedabad, IND; 3 Emergency Medicine, Smt. B. K. Shah Medical Institute & Research Centre, Vadodara, IND; 4 Medicine, Liaquat University of Medical and Health Sciences, Jamshoro, PAK; 5 Internal Medicine, Cavan General Hospital, Cavan, IRL; 6 Trauma and Orthopedics, Central Middlesex Hospital, London, GBR; 7 General Medicine, Abbasi Shaheed Hospital, Karachi, PAK; 8 General Medicine, Cavan Monaghan General Hospital, Cavan, IRL; 9 General Surgery, British United Provident Association, London, GBR; 10 Medicine, United States Navy, United States Military, North Chicago, USA; 11 Clinical Research, Arizona State University, Tempe, USA; 12 Internal Medicine, Shri B. M. Patil Medical College Hospital and Research Centre, Vijayapura, IND; 13 General Surgery, Abbasi Shaheed Hospital, Karachi, PAK

**Keywords:** apache-ii, mannheim peritonitis index, perforated viscus, possum, risk stratification, scoring systems

## Abstract

Perforated viscus remains a high-risk surgical emergency with significant mortality despite advances in perioperative care. Accurate risk stratification is essential to guide triage, surgical decision-making, and postoperative management. The available scoring tools are the Boey score (three-variable risk model: shock, comorbidity, delay >24 hours), Peptic Ulcer Perforation (PULP) score, Mannheim Peritonitis Index (MPI), Physiological and Operative Severity Score for the Enumeration of Mortality and Morbidity (POSSUM), and Acute Physiology and Chronic Health Evaluation II (APACHE-II), each addressing different dimensions of patient risk, ranging from physiological stability to intra-abdominal severity. Simpler models such as Boey and MPI are valuable in emergency and resource-limited settings due to their bedside applicability. In contrast, PULP and APACHE-II offer greater precision by integrating systemic comorbidities and biochemical indices, making them more suitable for tertiary centers. POSSUM provides a balanced approach by combining physiology with operative severity, though it may overestimate mortality. No single model is universally applicable, highlighting the need for context-specific use. Future research should focus on hybrid scoring systems that merge clinical simplicity with modern predictive analytics for broader global relevance.

## Introduction and background

Perforated viscus is a surgical emergency characterized by full-thickness disruption of the gastrointestinal wall, leading to contamination of the peritoneal cavity. It most commonly arises from perforated peptic ulcer disease, typhoid-related ileal perforations, diverticular disease, or malignancy-related colonic perforations. The ensuing peritonitis rapidly progresses to systemic inflammatory response syndrome and septic shock if not treated promptly. Despite significant advancements in diagnostic imaging, resuscitation, anesthesia, and surgical techniques, perforated viscus remains associated with substantial morbidity and mortality worldwide, with reported mortality ranging from 10% to 40% depending on site, delay in presentation, and patient comorbidities [1,2]. The burden of perforated viscus is compounded in resource-limited settings, where delays in presentation and limited access to emergency surgical services contribute to worse outcomes. Even in high-resource centers, mortality remains high among elderly and comorbid patients, reflecting the complex interplay between host factors, disease severity, and surgical urgency.

The heterogeneity of presentation, from contained localized perforations to diffuse generalized peritonitis, makes early stratification of risk crucial. Effective risk prediction not only guides triage decisions but also assists in optimizing preoperative resuscitation, informing surgical strategy, and providing realistic prognostic counseling to patients and families [3,4]. Over the past three decades, a range of scoring systems has been introduced to address this clinical need. Some, such as the Boey score and Mannheim Peritonitis Index (MPI), were specifically designed for perforation-related peritonitis and emphasize factors like shock, delay in presentation, and extent of contamination. Others, such as the Acute Physiology and Chronic Health Evaluation II (APACHE-II) and Physiological and Operative Severity Score for Enumeration of Mortality and Morbidity (POSSUM), were originally developed for critical care or general surgical patients but have since been validated in cohorts with perforated viscus.

More recently, the Peptic Ulcer Perforation (PULP) score has incorporated modern risk determinants, including immunosuppression, malignancy, and renal dysfunction, reflecting the evolving patient demographic. These tools vary in complexity, required variables, and predictive accuracy, which has limited their universal adoption [5,6]. The absence of a universally applicable scoring system creates a gap in clinical practice, where physicians often rely on clinical judgment rather than validated models. Differences in performance across populations also raise questions about generalizability and external validity. A systematic appraisal of the available tools is therefore warranted, particularly given the continued global burden of perforated viscus. This review systematically evaluates the evidence on risk stratification tools and scoring systems in perforated viscus, with the aim of identifying their strengths, limitations, and applicability to modern surgical practice, while highlighting areas for future research and innovation.

## Review

Materials and methods

Search Strategy

This systematic review was performed in accordance with the Preferred Reporting Items for Systematic reviews and Meta-Analyses (PRISMA) 2020 guidelines to ensure methodological rigor and transparency [7]. A structured protocol was developed before initiation, and two independent reviewers carried out the literature search, study selection, and data extraction processes. Any disagreements were resolved through discussion, with arbitration by a third reviewer when necessary. A comprehensive search of PubMed/MEDLINE, Embase, Scopus, and the Cochrane Library was conducted up to August 2025. The search strategy used a combination of MeSH terms and free-text keywords such as “perforated viscus”, “hollow viscus perforation”, “peritonitis”, “risk stratification”, “scoring system”, and “mortality prediction”. Boolean operators were employed to optimize sensitivity and specificity. Searches were restricted to human studies in the English language to ensure clinical relevance.

Eligibility Criteria

Eligibility was determined using the PICO framework [8]. The Population (P) was defined as adult patients (>18 years) undergoing surgery for a perforated viscus of any gastrointestinal origin. The Intervention (I) included the application of a risk stratification tool or scoring system in the perioperative setting. The Comparator (C) involved other validated scoring systems or clinical outcome measures. The Outcomes (O) of interest were postoperative mortality, morbidity, and predictive accuracy of the tools. Studies were included if they were original research articles with more than 40 patients, involved surgical management of perforated viscus, reported mortality or morbidity outcomes, and applied a validated scoring system. Exclusion criteria comprised case reports, non-English publications, pediatric studies, and animal experiments. This approach ensured that only clinically meaningful and generalizable evidence was synthesized.

Study Selection

All retrieved records underwent independent screening of titles and abstracts by two reviewers to assess initial relevance. Articles that appeared eligible were retrieved in full text and evaluated against the inclusion and exclusion criteria. Discrepancies between reviewers were resolved by consensus, with a third reviewer consulted when necessary. The entire process was documented using a PRISMA flow diagram to ensure transparency.

Data Extraction

Data were extracted independently by both reviewers using a standardized pro forma. Extracted information included study characteristics, population details, intervention or scoring tool applied, comparators, outcomes measured, and key findings. Any inconsistencies were cross-checked and resolved by discussion to maintain data accuracy and reduce bias.

Risk of Bias Assessment

The methodological quality of included studies was appraised using the Newcastle-Ottawa Scale for observational multicenter cohort studies [9]. These tools evaluated domains including study design, selection bias, comparability of cohorts, and outcome assessment. This structured approach ensured a robust assessment of potential biases that could influence the validity of findings.

Data Synthesis

Given the heterogeneity in study design, patient populations, and reported outcomes, a narrative synthesis was undertaken. The performance of different risk stratification tools was compared using measures of discriminative accuracy such as the receiver operating characteristic curve and area under the curve (AUC), along with sensitivity and specificity. This qualitative synthesis provided a balanced overview of the predictive capabilities of the available scoring systems.

Results

Study Selection Process

**Figure 1:**
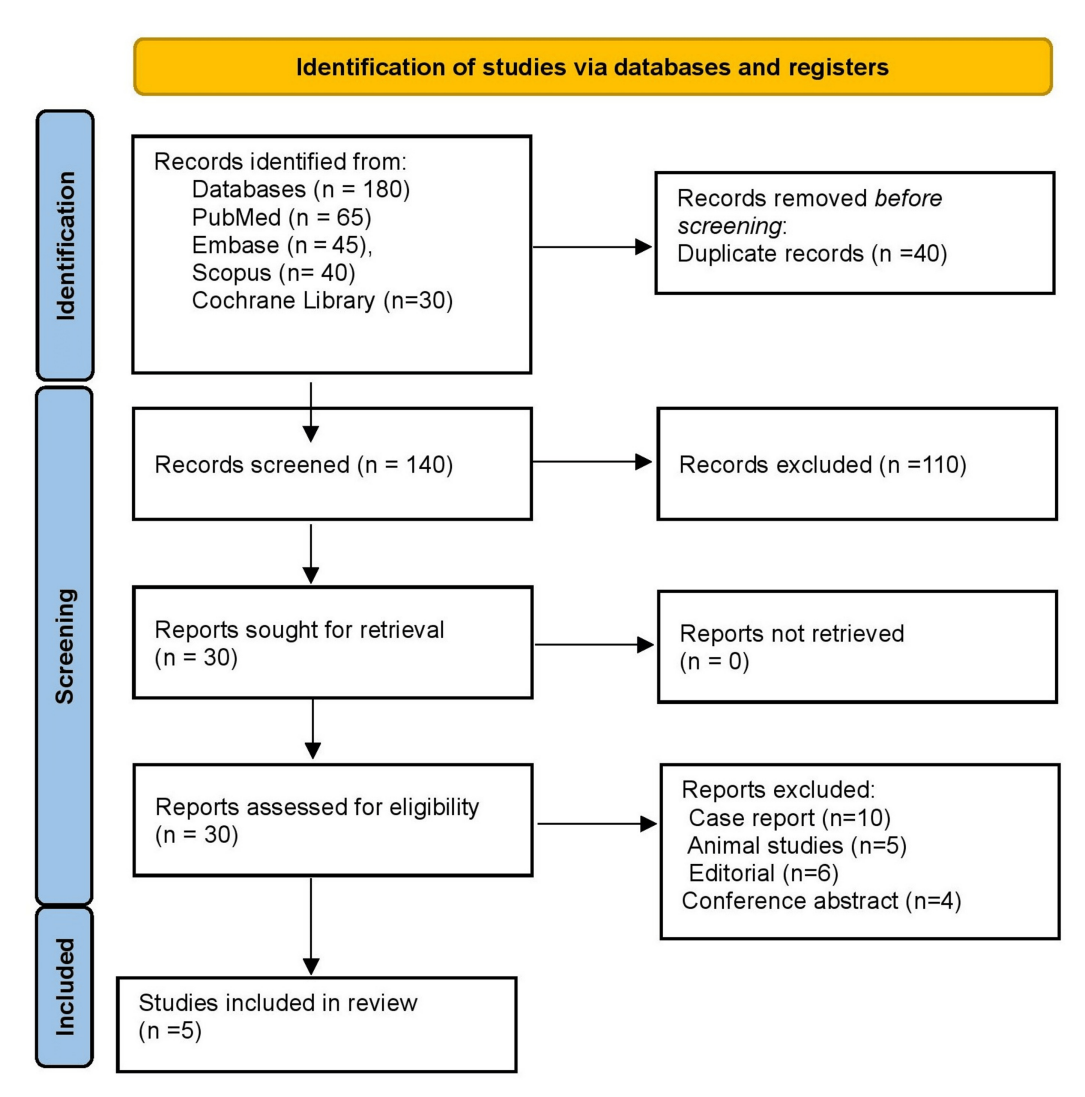
PRISMA flow diagram PRISMA, Preferred Reporting Items for Systematic reviews and Meta-Analyses

Figure 1 demonstrates 180 identified records (PubMed n = 65, Embase n = 45, Scopus n = 40, Cochrane n = 30); 40 duplicates were removed. After screening 140 abstracts, 110 were excluded for irrelevance. Thirty full-text reports were retrieved; 25 were excluded (10 case reports, five animal studies, six editorials, and four conference abstracts). Five studies met final inclusion, encompassing 3,127 patients.

Characteristics of the Selected Studies

Table 1 demonstrates the characteristics of selected studies, including Boey et al. (1987), who studied 259 duodenal ulcer perforations, showing mortality rising from 0% to 100% with increasing Boey risk factors (shock, comorbidity, delay >24 hours) [10]. Møller et al. (2012) validated the PULP score in 2,668 patients, achieving a strong 30-day mortality prediction (AUC 0.83) and integrating systemic dysfunction and comorbidities [11]. Sharma et al. (2015) assessed 100 laparotomy cases, with the MPI yielding high sensitivity and specificity; mortality was linked to age, organ failure, and non-colonic perforations [12]. Chatterjee and Renganathan (2015) used POSSUM in 50 patients, showing accurate calibration, with delay to surgery and comorbidity affecting outcomes [13]. Kulkarni et al. (2007) applied APACHE-II in 50 perforation cases, demonstrating excellent discrimination (AUC 0.984), highlighting systemic inflammatory burden and organ dysfunction [14].

**Table 1:** Characteristics of the selected studies ASA, American Society of Anesthesiologists; AUC, area under the curve; MPI, Mannheim Peritonitis Index; O:E ratio, observed-to-expected ratio; PPU, perforated peptic ulcer; ROC, receiver operating characteristic curve

Author and year	Population (P)	Exposure/condition (I)	Comparator (C)	Outcomes (O)	Pathophysiological findings	Anatomical impact	Risk stratification tools/scoring systems
Boey et al. (1987) [10]	259 consecutive adults undergoing surgery for a perforated duodenal ulcer	Perforated duodenal ulcer requiring operative repair	Boey risk-factor tiers: 0 vs. 1 vs. 2 vs. 3 predictors	Postoperative mortality stratified by count of risk factors (0%, 10%, 45.5%, and 100%)	Pre-op shock, major comorbidity, and perforation >24 hours reflect systemic inflammatory burden and physiological reserve	Duodenal (pyloroduodenal) perforation with generalized peritonitis in many cases	Boey score (three-variable risk model: shock, major illness, delay >24 hours)
Møller et al. (2012) [11]	2,668 patients from 35 Danish hospitals, surgically treated for gastric or duodenal PPU	Perforated peptic ulcer (gastric/duodenal), operative management	Compared the new PULP model vs. Boey and ASA	30-day mortality prediction (AUC 0.83); 27% overall 30-day mortality	Incorporates shock and organ dysfunction (elevated creatinine), immune/host deficits (steroids, malignancy, and cirrhosis), and time to admission	Gastric or duodenal perforation leading to peritonitis	PULP score (age >65, malignancy/AIDS, cirrhosis, steroids, delay >24 hours to admission, shock, creatinine >130 μmol/L, ASA grade)
Sharma et al. (2015) [12]	100 adults operated for secondary peritonitis due to hollow-viscus perforation	Perforation peritonitis managed by laparotomy	MPI categories: <15 vs. 16-25 vs. >25	Mortality by MPI class; ROC sensitivity 92%, specificity 78%	Mortality associated with age >50, organ failure, and generalized peritonitis; non-colonic sources carried a higher risk	Predominantly ileal/small-bowel perforations; diffuse peritoneal contamination frequent	MPI
Chatterjee and Renganathan (2015) [13]	50 patients with perforative peritonitis (single-unit prospective series)	Perforation peritonitis undergoing emergency surgery	Observed vs. expected outcomes by POSSUM	Accurate calibration (O:E ≈1.0 for mortality/morbidity); factors like delay to surgery and comorbidity significant	Physiological derangement plus operative severity mirror sepsis burden and host response	Mixed sites (gastroduodenal, small bowel, colonic) with varying contamination	POSSUM (12 physiological + 6 operative variables; study also examined delay and comorbidity)
Kulkarni et al. (2007) [14]	50 adults with peritonitis from hollow-viscus perforation	Emergency laparotomy for perforated peritonitis	APACHE-II score bands and ROC analysis	AUC 0.984; score 11-20 best discriminated mortality risk	High APACHE-II reflects systemic inflammatory response, organ dysfunction, and hemodynamic instability	Mixed hollow-viscus perforations culminating in secondary peritonitis	APACHE-II (physiology-based ICU severity model applied pre-op)

Risk of Bias Assessment

Table 2 demonstrates that the risk of bias assessment of the study by Boey et al. (1987) was rated moderate risk due to limited variables and its single-center design [10]. Møller et al. (2012) achieved a low-risk rating, supported by its large multicenter dataset [11]. Sharma et al. (2015) was considered a moderate risk owing to its small single-center population [12]. Chatterjee and Renganathan (2015) were rated high risk because of their underpowered sample size [13]. Kulkarni et al. (2007) was judged a moderate risk, reflecting a modest sample size despite reasonable methodology [14].

**Table 2:** Risk of bias assessment NOS, Newcastle-Ottawa Scale

Study	Study design	Tool used	Risk of bias rating	Justification
Boey et al. (1987) [10]	Prospective cohort	NOS	Moderate	Limited variables, single-center
Møller et al. (2012) [11]	Multicenter cohort	NOS	Low	Large sample, multicenter
Sharma et al. (2015) [12]	Prospective cohort	NOS	Moderate	Small sample, single-center
Chatterjee and Renganathan (2015) [13]	Prospective	NOS	High	Small, underpowered
Kulkarni et al. (2007) [14]	Prospective	NOS	Moderate	Modest sample size

Discussion

Perforated viscus continues to pose a formidable challenge in surgical practice, with mortality remaining high despite advances in perioperative care. The pathological cascade begins with leakage of gastrointestinal contents into the peritoneal cavity, triggering a rapid inflammatory response and systemic sepsis. Progression to shock and multi-organ dysfunction is common when diagnosis or intervention is delayed. Against this background, risk stratification tools provide objective frameworks to estimate prognosis, prioritize operative timing, and guide the intensity of postoperative monitoring. Their importance lies in supplementing clinical judgment with quantifiable predictors that can capture the complex interplay between host reserve, disease severity, and surgical burden.

The Boey score, though simple, addresses three critical prognostic dimensions: physiological shock, underlying systemic illness, and duration of perforation. Each reflects a surrogate for physiological reserve, sepsis severity, and surgical delay. However, its limited variable set reduces discriminatory power in heterogeneous populations such as elderly patients with multiple comorbidities [10]. The PULP score overcomes some of these limitations by integrating biochemical derangements (elevated creatinine), immunosuppression (steroid use, malignancy, and cirrhosis), and ASA grading into its model. This broader scope enhances predictive accuracy, particularly in populations where chronic disease modifies host response to sepsis [11]. The MPI is distinctive in incorporating intra-abdominal contamination variables. By assigning weight to the extent and character of peritoneal contamination, as well as the source of perforation, MPI reflects the pathophysiological severity of peritonitis. Its ability to stratify patients into low, intermediate, and high-risk categories makes it especially useful in acute emergency laparotomies where intraoperative findings strongly influence outcomes [12].

Similarly, the POSSUM score combines 12 physiological and six operative parameters, integrating preoperative patient status with the technical severity of surgery. While sometimes criticized for overestimating mortality, its strength lies in providing a holistic risk assessment across surgical domains [13]. The APACHE-II score represents a more intensive care-oriented model, capturing systemic inflammatory and organ dysfunction variables such as arterial pH, oxygenation, serum creatinine, and hemodynamic status. Its high AUC in predicting mortality underscores the importance of systemic physiology in risk determination. However, the complexity and requirement for laboratory values make it less practical for bedside surgical decision-making in resource-limited or emergent contexts [14]. In contrast, models like Boey or MPI, though less comprehensive, retain utility for rapid triage and surgical prioritization.

Taken together, these scoring systems underscore that risk prediction in perforated viscus is multi-dimensional, requiring integration of physiological stability, comorbid illness, biochemical status, and operative severity. No single tool achieves universal applicability, but their combined insights help tailor decision-making to the clinical context. Simpler bedside models may suffice in frontline emergency settings, while advanced systems like PULP or APACHE-II are better suited to tertiary care centers with wider diagnostic capabilities [15]. This review emphasizes that a stratified approach to risk prediction, guided by available tools, can improve surgical outcomes and optimize resource utilization. The review is constrained by heterogeneity in study designs and regional variations, with most data originating from single-center experiences, limiting external validity and global applicability.

## Conclusions

Perforated viscus remains a high-risk surgical emergency with significant mortality despite advances in perioperative care. Accurate risk stratification is essential to guide triage, surgical decision-making, and postoperative management. The available scoring tools, such as Boey, PULP, MPI, POSSUM, and APACHE-II, each address different dimensions of patient risk, ranging from physiological stability to intra-abdominal severity. Simpler models, such as Boey and MPI, are particularly valuable in emergency and resource-limited settings due to their bedside applicability. In contrast, PULP and APACHE-II offer greater precision by incorporating systemic comorbidities and biochemical indices, making them more suitable for tertiary care centers. POSSUM provides a balanced approach by combining physiological and operative severity, though it may overestimate mortality. No single model is universally applicable, highlighting the need for context-specific use. Future research should focus on hybrid scoring systems that integrate clinical simplicity with modern predictive analytics for broader global applicability.

## References

[1] Svanes C (2000). Trends in perforated peptic ulcer: incidence, etiology, treatment, and prognosis. World J Surg.

[2] Møller MH, Shah K, Bendix J, Jensen AG, Zimmermann-Nielsen E, Adamsen S, Møller AM (2009). Risk factors in patients surgically treated for peptic ulcer perforation. Scand J Gastroenterol.

[3] Søreide K, Thorsen K, Søreide JA (2014). Strategies to improve the outcome of emergency surgery for perforated peptic ulcer. Br J Surg.

[4] Freeman HJ (2014). Spontaneous free perforation of the small intestine in adults. World J Gastroenterol.

[5] Gupta S, Kaushik R (2006). Peritonitis - the Eastern experience. World J Emerg Surg.

[6] Thorsen K, Søreide JA, Søreide K (2013). Scoring systems for outcome prediction in patients with perforated peptic ulcer. Scand J Trauma Resusc Emerg Med.

[7] Page MJ, McKenzie JE, Bossuyt PM (2021). Updating guidance for reporting systematic reviews: development of the PRISMA 2020 statement. J Clin Epidemiol.

[8] Eldawlatly A, Alshehri H, Alqahtani A, Ahmad A, Al-Dammas F, Marzouk A (2018). Appearance of Population, Intervention, Comparison, and Outcome as research question in the title of articles of three different anesthesia journals: a pilot study. Saudi J Anaesth.

[9] Wells GA, Shea BJ, O'Connell D, Peterson J, Welch V, Losos M, Tugwell P (2000). The Newcastle-Ottawa Scale (NOS) for Assessing the Quality of Non-Randomized Studies in Meta-Analysis. https://www.researchgate.net/publication/261773681_The_Newcastle-Ottawa_Scale_NOS_for_Assessing_the_Quality_of_Non-Randomized_Studies_in_Meta-Analysis.

[10] Boey J, Choi SK, Poon A, Alagaratnam TT (1987). Risk stratification in perforated duodenal ulcers. A prospective validation of predictive factors. Ann Surg.

[11] Møller MH, Engebjerg MC, Adamsen S, Bendix J, Thomsen RW (2012). The Peptic Ulcer Perforation (PULP) score: a predictor of mortality following peptic ulcer perforation. A cohort study. Acta Anaesthesiol Scand.

[12] Sharma R, Ranjan V, Jain S, Joshi T, Tyagi A, Chaphekar R (2015). A prospective study evaluating utility of Mannheim peritonitis index in predicting prognosis of perforation peritonitis. J Nat Sci Biol Med.

[13] Chatterjee AS, Renganathan DN (2015). POSSUM: a scoring system for perforative peritonitis. J Clin Diagn Res.

[14] Kulkarni SV, Naik AS, Subramanian N Jr (2007). APACHE-II scoring system in perforative peritonitis. Am J Surg.

[15] Marsh HM, Krishan I, Naessens JM (1990). Assessment of prediction of mortality by using the APACHE II scoring system in intensive-care units. Mayo Clin Proc.

